# Integrating multi-omics and machine learning to unravel mechanisms of lymph node metastasis in papillary carcinoma with and without thyroiditis

**DOI:** 10.3389/fimmu.2026.1708330

**Published:** 2026-02-18

**Authors:** Shuran Chen, Wencan Yang, Yanyan Liu, Angqing Li

**Affiliations:** Department of General Surgery, The Third Affiliated Hospital of Anhui Medical University (The First People’s Hospital of Hefei), Hefei, China

**Keywords:** lymph node metastasis, machine learning, papillary thyroid carcinoma, thyroiditis, tumor microenvironment

## Abstract

**Background:**

Papillary thyroid carcinoma (PTC) is the most common thyroid malignancy, with lymph node metastasis (LNM) significantly influencing prognosis. PTC cases were categorized based on the presence or absence of coexisting thyroiditis: those with thyroiditis were designated as PTC-thyroiditis, and those without as PTC-blank. Although both subtypes exhibit distinct clinical and genomic profiles, the mechanisms underlying LNM—particularly the role of extracellular matrix (ECM) remodeling and cancer-associated fibroblasts (CAFs)—remain poorly understood. This study aimed to compare the molecular and cellular heterogeneity of PTC-blank and PTC-thyroiditis, identify key drivers of LNM, and develop a predictive model for clinical use.

**Methods:**

We integrated bulk RNA-seq data from the TCGA database and single-cell RNA-seq (scRNA-seq) data from GSE184362 to analyze clinical, genomic, and tumor microenvironment (TME) differences between PTC-blank and PTC-thyroiditis. Differential expression analysis, Gene Set Enrichment Analysis (GSEA), and immune cell infiltration analysis were performed. Fibroblast subpopulations were characterized at single-cell resolution. Machine learning algorithms (LASSO, random forest, KNN) were applied to identify LNM-related genes and construct a predictive model. Mendelian randomization (MR) and molecular docking were used to validate causal genes and potential drug interactions.

**Results:**

PTC-blank exhibited higher T, N, and M stages and increased mutations in BRAF and MUC16 compared to PTC-thyroiditis. LNM in PTC-blank was associated with ECM remodeling and collagen fiber accumulation, associated with a distinct PI16+ fibroblast subcluster with active ECM organization functions. In contrast, LNM in PTC-thyroiditis involved immune-related pathways without significant fibroblast infiltration or ECM changes. A 17-gene predictive model for LNM was developed, with the KNN classifier demonstrating high accuracy. MR analysis identified SHISA5 as a causal risk gene for thyroid cancer, and molecular docking revealed strong binding affinity with acetaminophen, suggesting therapeutic potential.

**Conclusions:**

PTC-blank and PTC-thyroiditis exhibit distinct LNM mechanisms: ECM remodeling and fibroblast infiltration are associated with metastasis in PTC-blank, while immune dysregulation appears to be more prominent in PTC-thyroiditis. The identified 17-gene model offers robust predictive value for LNM risk, and SHISA5 represents a novel causal gene and potential therapeutic target. These findings provide insights into subtype-specific management strategies for PTC patients.

## Introduction

Papillary thyroid carcinoma (PTC) is the most common type of thyroid carcinoma (THCA), and has become an important health issue in recent years due to its increasing incidence worldwide (1, 2). Its prognosis is generally favorable; however, many patients experience lymph node metastasis (LNM) (3). The process of PTC-related LNM (PTC-LNM) is closely associated with local recurrence, distant metastasis, and reduced survival rates (4). Central and lateral neck LNM are independent prognostic factors for predicting disease-free survival (DFS) in patients with PTC (5). Therefore, it is crucial to elucidate the underlying mechanisms that influence PTC-LNM.

Genetic damage is the “match that lights the fire” of cancer, while certain types of inflammation may serve as the “fuel that fans the flames.” (6) Inflammatory factors play a significant role in developing PTC-LNM. Studies have shown that pre-treatment systemic immune-inflammatory response index (SII), systemic inflammatory response index (SIRI), and other inflammatory markers were independent risk factors for LNM in patients with PTC. Thyroiditis is a common autoimmune disease characterized by chronic inflammation of the thyroid gland and reduced thyroid function (7). Recently, growing evidence has highlighted a significant correlation between thyroid inflammation, especially Hashimoto’s Thyroiditis (HT), and the pathological progression of PTC (8). According to the results of a meta-analysis that included multiple studies, HT has been confirmed as an independent risk factor for PTC development. Moreover, patients with PTC and HT have a higher incidence of multifocality. Notably, these patients exhibit unique biological behavior during disease progression, with significantly lower rates of extrathyroidal invasion and regional LNM than patients with PTC alone (9). A retrospective cohort study further supported this phenomenon, showing that patients with coexisting HT had lower clinical aggressiveness and significantly higher 10-year DFS rate. This suggests that this specific subtype may have unique tumor microenvironment (TME) characteristics that affect disease outcomes (10). However, the precise mechanisms between PTC with or without Thyroiditis during LNM remain unclear.

The aim of this study was to elucidate the different features in PTC with or without Thyroiditis during LNM by integrating RNA-seq and single-cell RNA-seq (scRNA-seq) data. Bulk RNA-Seq is a technique for studying gene expression. It elucidates the relationship between changes in gene expression and the occurrence and development of tumors by analyzing the transcriptional expression of all cells in a tissue (11). scRNA-seq allows researchers to analyze gene expression at the single-cell level (12–14). Integrating bulk transcriptome sequencing and scRNA-seq explores molecular changes at the overall level, captures cellular heterogeneity, and reveals the transcriptional characteristics of different cell types (15). For example, a study used combined transcriptome sequencing data and scRNA-seq to reveal the molecular characteristics of thyroid dysfunction between patients with PTC and HT and those receiving immunotherapy (16). After clarifying the molecular differences and cellular heterogeneity between these patient groups using bulk RNA-Seq and scRNA-seq, we constructed diagnostic models for PTC-LNM based on these molecules using various machine learning algorithms to identify potential biomarkers that may guide clinical decision-making and personalized treatment strategies for patients with PTC.

In summary, by employing various research methods, we aimed to address the existing knowledge gap regarding the interactions between thyroiditis and PTC. This study’s findings could significantly enhance our understanding of the crosstalk between thyroiditis and PTC, potentially paving the way for innovative therapeutic strategies tailored to individual patient characteristics, thereby optimizing the clinical management of this increasingly prevalent cancer.

## Materials and methods

### Acquisition of clinical data

Formalin-fixed, paraffin-embedded tissue blocks were obtained from The first people’s Hospital of Hefei. The project was approved by Ethics Committee of the First People’s Hospital of Hefei. Postoperative pathological staging was performed according to the American Joint Committee on Cancer (AJCC) 8th edition staging system (17).

### Data acquisition and sorting of transcriptome and microarray data

RNA-seq data and clinical information for patients with thyroid cancer were collected from UCSC Xena (18), including 260 PTC tissues without thyroiditis (PTC_blank) and 62 PTC tissues with thyroiditis (PTC_thyroiditis) for further analyses (**Supplementary Table 1**). Microarray data were obtained from the Gene expression Omnibus (GEO) database. The GSE138198 (19) dataset included microarray data for six PTC-blank cases and eight cases of PTC-thyroiditis. The GSE60542 (20) dataset comprised microarray data for 30 normal thyroid tissues, 14 PTC tissues (N0), 19 PTC tissues (N1), and 23 LNM tissues. The tinyArray package was used to convert probe names into gene symbols, and only genes expressed in more than 50% of the samples were retained for subsequent analysis.

### Acquisition, filtering, and cell annotation of scRNA

Single-cell transcriptomic data were downloaded from GSE184362 (21). From the GSE184362 dataset, we downloaded single-cell sequencing data from 12 patients (including six normal adjacent tissues and six tumor tissues). We retained the genes and cells that met the following criteria: ① unique molecular identifier (UMI) counts < 6000; ② 200 < nFeature_RNA < 5000; ③ mitochondrial gene expression below 30%. Highly variable genes in the normalized data were identified using FindVariableFeatures. After performing principal component analysis (PCA), we used harmony to remove batch effects between samples and then performed dimensionality reduction using Uniform Manifold Approximation and Projection (UMAP). Cells were clustered using FindClusters, and each cluster was manually annotated based on marker genes from previous studies. The UMAP plots were visualized using the scRNAtoolVis and scCustomize packages.

### AUCell and cell–cell communication analysis

Highly expressed genes in each cell cluster were identified using FindAllMarkers (min.pct = 0.1, logfc.threshold = 0.1). The top 5% of genes from each cell cluster were used for functional and pathway scoring with the AUCell software. The scoring threshold was calculated using AUCellExplore thresholds, and cells were categorized into High and Low groups based on this threshold. Cell communication analysis was conducted using CellChat software (22), which includes a comprehensive database of signaling molecule interactions, and considers the established structural characteristics of receptor-ligand interactions. This includes various forms of multimeric receptor-ligand complexes, soluble agonists, antagonists, and stimulatory and inhibitory membrane-bound coreceptors.

### Clinical and genomic characteristics of patients with PTC

TNM staging and stage data were collected from patients, and ggplot2 was used to draw a pie chart of the different proportions. Patient survival status and survival time data were collected, and the Survminer package was used to construct the Kaplan–Meier (K-M) plot. The fviz_pca_ind function was used to construct the PCA plots. Mutation information of patients with THCA was obtained using the Cancer Genome Atlas (TCGA) mutations package, and mutation waterfall plots were drawn using the maftools package. Previous studies have suggested that the Thyroid Differentiation Score (TDS) is associated with LNM and poor prognosis in patients with PTC (23, 24). We collected data on 13 genes (TG, TPO, SLC26A4, DIO2, TSHR, PAX8, DUOX1, DUOX2, NKX2-1, GLIS3, FOXE1, TFF3, FHL1) to calculate the TDS.

### Differential expression, enrichment analysis, correlation analysis, and immune infiltration analysis

Differential expression analysis of the microarray data was performed using the limma package to identify differentially expressed genes (DEGs) between the PTC-blank and PTC-thyroiditis groups. The Differential Expression analysis based on the Negative Binomial Distribution (version 2) (DESeq2) package was used to conduct differential expression analysis of TCGA transcriptome data to identify DEGs associated with LNM in PTC-blank and PTC-thyroiditis. Gene Set Enrichment Analysis (GSEA) was performed on differential analysis results using the clusterProfiler and msigdbr (Homo sapiens_C5_BP and Homo sapiens_C2_REACTOME). The ggplot2 package was used to visualize the top five entries with the highest normalized enrichment scores (NES) from each GSEA enrichment analysis result. Gene ontology (GO) and Kyoto Encyclopedia of Genes and Genomes (KEGG) analyses were conducted using enrichGO and enrichKEGG, respectively, and the top five entries with the highest z-scores were presented using ggplot2. Correlation analysis was performed using the LinkET software. The immune infiltration status of the PTC-LNMs was assessed using CIBERSORT, MCPcounter, and EPIC.

### Weighted gene co-expression network analysis and Mfuzz analysis

Detailed introductions to the Weighted Gene Co-expression Network Analysis (WGCNA) and Mfuzz analyses are described in previous literature (25, 26). For WGCNA, the top 50% of genes showing the greatest variance in GSE60542 were selected to identify the gene co-expression modules associated with PTC-LNM. The pickSoftThreshold function was used to select the optimal soft threshold. The blockwiseModules function was employed to construct a network with at least 30 genes per module. A heat map illustrating the correlation between traits and modules was generated, and the genes from the module with the highest correlation were selected for further analysis. Based on the TCGA transcriptome data, clustering analysis was performed using the Mfuzz package. After selecting the optimal number of clusters, line plots and cluster heat maps were constructed using ClusterGVis.

### Construction and verification of the predictive PTC-LNM model

To identify core genes associated with LNM in papillary thyroid carcinoma (PTC), we employed LASSO (Least Absolute Shrinkage and Selection Operator) regression for feature selection. Using RNA-seq expression data from PTC samples in the TCGA database, the expression matrix was normalized and log-transformed by log_2_(CPM + 1), and a candidate gene set obtained from prior analyses was extracted. With LNM status (N0/N1) as the binary dependent variable and the gene expression matrix as the independent variable, LASSO regression was performed using the glmnet R package. The optimal regularization parameter λ (λ_min) was determined through 10-fold cross-validation, and features were selected based on the criterion of minimizing the mean squared error. Similarly, a random forest model was constructed using the randomForest R package to screen for core genes related to PTC LNM, and the top 20 genes ranked by importance were visualized. Additionally, edgeR and the Combat package were used to perform log transformation and normalization of the TCGA and GSE60542 data to correct for batch effects. In the TCGA database, we first classified the sample into PTC-blank and PTC-thyroiditis groups. Subsequently, using the createDataPartition function, the PTC-blank samples were randomly divided into a training and a testing set at a ratio of 7:3. Eight machine-learning algorithms (27), including logistic regression (LR), decision tree (DT), random forest (RF), K-nearest neighbor (KNN), support vector machine (SVM), extreme gradient boosting (XGB), stochastic gradient boosting (SGBT), and neural network (NNET) were used to train a 17-gene model. PTC-thyroiditis samples were used as test set 1, while samples from GSE60542 were used as test set 2 to validate the model. The e model’s reliability was evaluated using the Receiver Operating Characteristic (ROC) curve. Confusion matrices were used to illustrate the relationship between the model’s predicted results and actual outcomes. Calibration curves were assessed to reflect the match between the predicted probabilities and actual results. Decision Curve Analysis (DCA) was employed to determine the model’s net benefit across different thresholds. We employed multiple statistical methods to evaluate the calibration performance of the models. The accuracy of predicted probabilities was quantified using the Brier score (mean squared error) and expected calibration error (ECE, based on 10 probability bins), and statistical testing was performed using the Spiegelhalter z-test. The 95% confidence intervals for all metrics were calculated through 1,000 bootstrap resampling iterations. The analysis encompassed the training set, an independent test set, and an external validation set to ensure comprehensive assessment. Calibration curves were visualized to intuitively illustrate the agreement between predicted probabilities and observed frequencies.

### Mendelian randomization analysis

This research utilized a Mendelian randomization (MR) approach to examine potential causal associations between 17 candidate genes and thyroid cancer risk. The investigation positioned these genes as exposure variables while considering thyroid cancer as the outcome measure. Genetic instrument selection involved stringent criteria: single nucleotide polymorphisms (SNPs) were extracted from eQTLGen Consortium’s expression quantitative trait loci (eQTL) data for the target genes, while thyroid cancer genome-wide association study (GWAS) data were obtained from the IEU OpenGWAS repository. Instrumental variable selection was performed using the TwoSampleMR package (version 0.5.7), implementing a genome-wide significance threshold (P < 1×10^-6) to ensure strong exposure associations. Linkage disequilibrium (LD) pruning was conducted with parameters set at r² < 0.01 within a 10,000 base pair window. Instrument strength was validated through F-statistics, with all selected instruments exceeding the recommended threshold of 10. Allelic alignment between exposure and outcome datasets was ensured by excluding palindromic SNPs and correcting for strand orientation. Causal estimation employed multiple MR methodologies, including inverse variance weighted (IVW), MR-Egger, weighted median, simple mode, and weighted mode approaches, with IVW serving as the primary analytical method. Statistical significance was defined as P < 0.05. Comprehensive sensitivity analyses were performed to validate result robustness, incorporating heterogeneity testing via Cochran’s Q statistic and pleiotropy assessment through MR-Egger regression intercept analysis (P < 0.05 considered significant). Potential outlier SNPs were identified using MR-PRESSO (version 1.0), and leave-one-out analysis evaluated the stability of effect estimates. The final analysis revealed statistically significant causal relationships between the 17 candidate genes and thyroid cancer susceptibility, with all sensitivity analyses supporting these findings. The comprehensive methodological approach strengthened the validity of the observed genetic associations.

### Molecular docking analysis

To evaluate the potential of hub genes as a therapeutic approach for thyroid cancer, we conducted molecular docking simulations between Acetaminophen and SHISA5. We obtained the atomic and molecular structure of SHISA5 from the Protein Data Bank (PDB, accessible at https://www1.rcsb.org/). PyMOL 2.3.0 successfully removed all interfering ligand precursors and water molecules present in the target. The molecular structure of Acetaminophen was extracted from the PubChem database (https://pubchem.ncbi.nlm.nih.gov/). Using Chem3D software, we performed dual modifications to the conformation of Acetaminophen at both the molecular and mechanical levels. Auto Dock Tools 1.5.6 was employed to dock the target protein molecule with SHISA5 after hydrogenation. A binding energy of less than -5 (kcal/mol) indicates a strong binding affinity between the small molecule and the protein.

### Immunohistochemical staining

Formalin-fixed, paraffin-embedded tissue blocks were fixed in 4% paraformaldehyde were baked at 60 °C for 1 h, dewaxed in xylene, and rehydrated in a graded series of ethanol solutions. Antigen retrieval was performed by microwaving the samples in 10 mM sodium citrate buffer (pH 6.0) for 15 min (5 min × 3 times), and the reaction was quenched with 3% hydrogen peroxide. After washing with Phosphate buffered saline, the samples were incubated overnight at 4 °C with anti-human Lysyl oxidase (LOX) antibody (Proteintech, 17958-1-AP, 1:50). Diaminobenzidine was used for detection. The images were quantitatively processed using the Analyze-Measure tool in ImageJ settings, based on the percentage of positively stained cells and staining intensity in representative sections for each field of view.

### Sirus red staining

The paraffin sections were dehydrated by placing them sequentially in xylene, anhydrous ethanol, and ethanol solutions with decreasing concentrations and finally rinsing with distilled water. Then, the sections were immersed in a saturated solution of Sirius Red (Ketubio, BL-RS-19) for staining, then washed in anhydrous ethanol for 8 minutes. After drying the sections in an oven at 60 °C, they were cleared in xylene and finally mounted with neutral gum. The stained sections were examined under an optical microscope, and the images were quantitatively processed using the Analyze-Measure tool in the ImageJ software.

### Statistical analyses

All statistical analyses were performed using R software (version 4.3.1). Continuous variables were compared between groups using the Wilcoxon rank-sum test, while categorical variables were analyzed using the Chi-square test or Fisher’s exact test as appropriate. Pearson’s correlation test was applied specifically to evaluate linear associations between continuous variables. Survival outcomes were assessed using Kaplan-Meier curves, with patients stratified into two groups based on clinical characteristics; differences in survival between groups were compared using the log-rank test. For predictive modeling, eight machine learning algorithms were implemented and evaluated using metrics including AUC, accuracy, sensitivity, and specificity. A p-value < 0.05 was considered statistically significant throughout the study.

## Results

### Clinical and genomic differences between PTC-blank and PTC-thyroiditis

**Figure 1:**
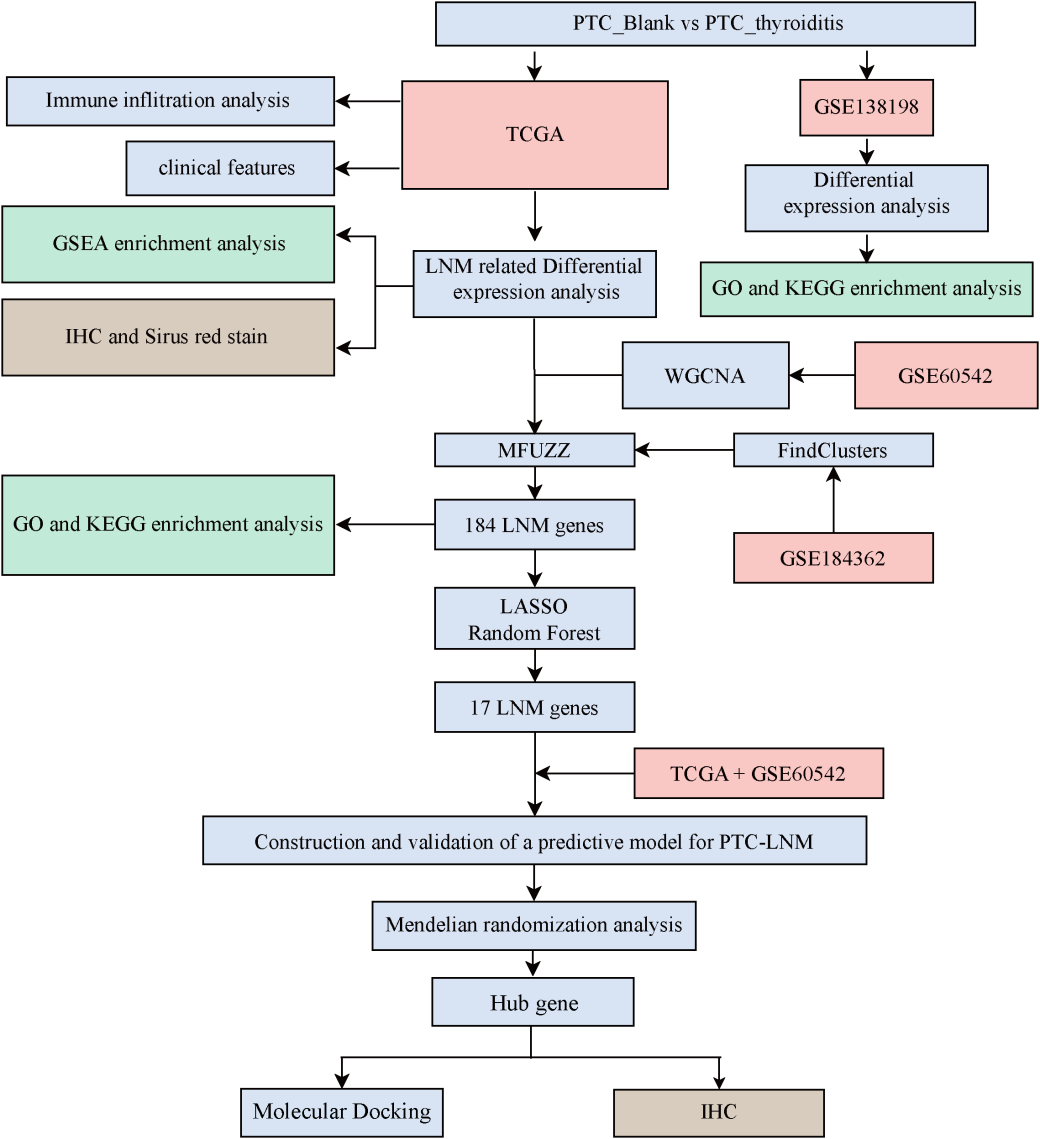
Schematic overview of the study.

**Figure 2:**
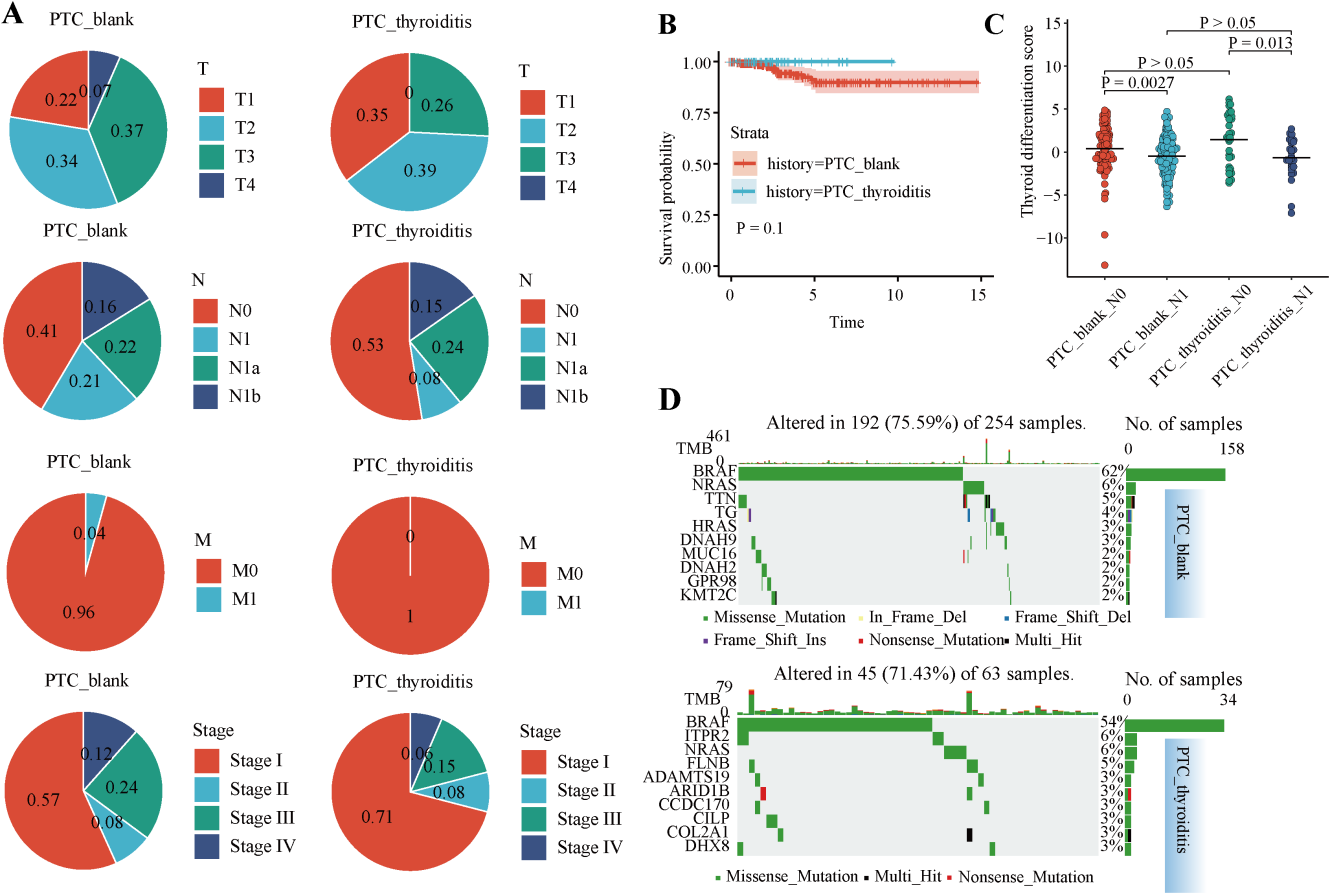
Clinical and genomic differences between PTC-blank and PTC-thyroiditis. **(A)** Pie charts show the distribution of sample counts across tumor stages for PTC-thyroiditis and PTC-blank patients. **(B)** Kaplan–Meier overall survival curves for PTC-thyroiditis and patients with PTC-blank. **(C)** Boxplots of TDS across the four groups. **(D)** Somatic mutation profiles of PTC-thyroiditis and patients with PTC-blank.

**Figure 1** presents the analytical workflow used in this study. We examined the distribution of clinical stages in the PTC-blank and PTC-thyroiditis samples to elucidate the impact of thyroiditis on the staging and prognosis of patients with PTC (**Figure 2A**). Regarding T, N, and M staging, PTC-blank tumors were associated with significantly more advanced stages compared to PTC-thyroiditis tumors. Compared with patients with PTC-thyroiditis, those with PTC-blank had a higher proportion of multiple pathological factors, including higher T stage (T3+T4 = 44%). The K-M curve of the difference in overall survival between the two patient groups was not statistically significant (**Figure 2B**). Both PTC-blank-LNM and PTC-thyroiditis-LNM groups showed a decrease in TDS (**Figure 2C**). At the genomic level, similar to patients with PTC-blank, the most common somatic mutated genes in patients with PTC-thyroiditis were the BRAF and NRAS genes. However, other mutations, such as TTN, TG, and MUC16 genes were observed in patients with PTC-blank, whereas no obvious mutations were observed in these genes in patients with PTC-thyroiditis (**Figure 2D**).

### Differences in ECM remodeling and fibroblast infiltration during LNM in PTC-blank and PTC-thyroiditis

**Figure 3:**
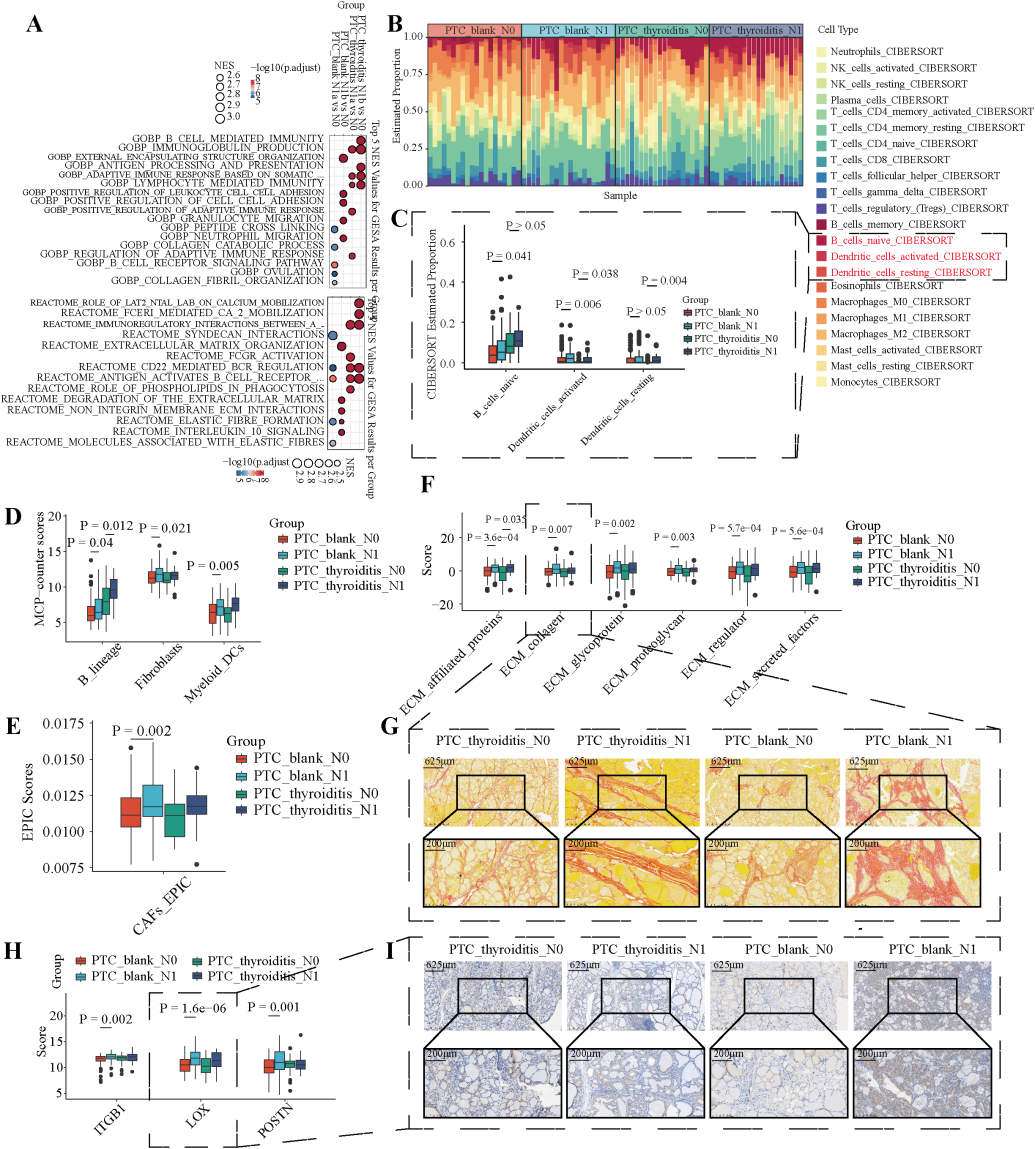
Differential functions and microenvironments in PTC-blank and PTC-thyroiditis with or without LNM. **(A)** GSEA enrichment results of GOBP (up) and REACTOME (down) pathways across the four groups. The top 5 NES items per group are shown. **(B)** The proportions of 22 immune cell subtypes calculated by CIBERSORT between PTC-blank and PTC-thyroiditis of different N stages in the TCGA-THCA cohort. **(C)** Boxplots of CIBERSORT-estimated proportions for three immune cell types across the four groups. **(D)** MCP-counter scores for three immune cell types between PTC-blank and PTC-thyroiditis of different N stage in the TCGA-THCA cohort. **(E)** EPIC scores for fibroblasts between PTC-blank and PTC-thyroiditis of different N stage in TCGA-THCA cohort. **(F)** ECM scores between PTC-blank and PTC-thyroiditis of different N stage in TCGA-THCA cohort. **(G)** Picrosirius red staining of PTC-thyroiditis tissue and PTC-blank in different stages of LNM. **(H)** The expression levels of three ECM-related genes between PTC-blank and PTC-thyroiditis of different N stage in the TCGA-THCA cohort. **(I)** The expression levels of LOX in PTC-thyroiditis tissue and PTC-blank in different stages of LNM.

Clinically, LNM in patients with PTC has received increasing attention. We further categorized PTC-blank and PTC-thyroiditis patients in the TCGA database based on the status of LNM into N0, N1a, and N1b. Differential expression analysis was conducted using DESeq2, and GSEA enrichment analysis of the functions and pathways was performed based on the differential analysis results (**Supplementary Table 2**). In patients with PTC-thyroiditis, we found that LNM was primarily associated with immune-related abnormalities, manifested as abnormal activation of functions and pathways, such as B_CELL_MEDIATED_IMMUNITY and CD22_MEDIATED_BCR_REGULATION (**Figure 3A**). Unlike PTC-thyroiditis, LNM in patients with PTC-blank is primarily associated with abnormalities in the extracellular matrix, manifested as abnormal activation of functions and pathways, such as COLLAGEN_CATABOLIC_PROCESS and EXTRACELLULAR_MATRIX_ORGANIZATION (**Figure 3A**). In the TME, fibroblasts and dendritic cells significantly impact the ECM composition of the extracellular matrix. Therefore, we calculated the infiltration levels of fibroblasts and dendritic cells at different LNM stages of PTC. **Figure 3B** shows the proportions of 22 immune cells in the tissues of patients with PTC at different metastasis stages. Based on CIBERSORT, MCPcounter, and EPIC, we found that PTC-blank-LNM showed a more pronounced increase in the infiltration of dendritic cells and fibroblasts, whereas there was no statistically significant difference in fibroblast infiltration in PTC-thyroiditis-LNM tissues (**Figures 3C-E**). Consistent with the GSEA enrichment analysis results, components such as collagen were significantly enriched in the PTC-blank-LNM tissues (**Figure 3F**). During LNM, the increase in collagen fiber content in the PTC-thyroiditis tissues was far lower than that in the PTC-blank group (**Figure 3G**). In addition, several molecules related to the regulation of extracellular matrix components were significantly upregulated in PTC-blank-LNM tissues, a phenomenon not observed in PTC-thyroiditis-LNM (**Figures 3H, I**). These results suggest that in patients with PTC-blank, the accumulation of collagen fibers is essential for LNM. In contrast, the accumulation of collagen fibers appears less prominent in PTC-thryroiditis-LNM, suggesting a potentially different mechanism.

### Identification of fibroblast subpopulations associated with ECM remodeling in PTC-blank

**Figure 4:**
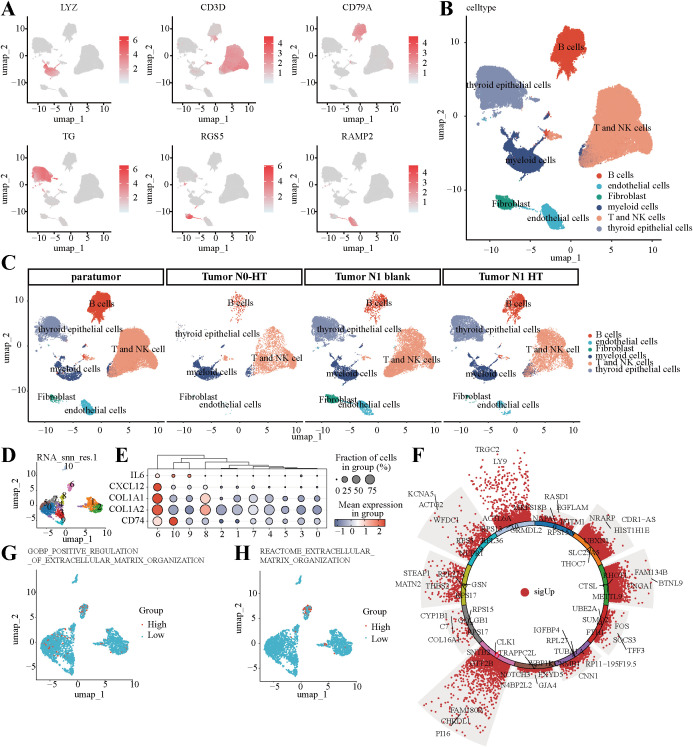
Identification of genes related to extracellular matrix remodeling in fibroblasts. **(A)** Expression levels of canonical marker genes for major cell lineages. **(B)** UMAP plot of all high-quality cells, color-coded by major cell lineage. **(C)** UMAP plot of all high-quality cells, color-coded by major cell lineage in adjacent non-tumor tissues and PTC tumor tissues across three histological groups (PTC-HT-N0, PTC-HT-N1, PTC-blank-N1). **(D)** UMAP plot of all fibroblasts, color-coded by 11 clusters identified at resolution 1.0. **(E)** Dotplot shows expression of five fibroblast-associated genes across 11 fibroblast clusters. **(F)** Volcano plot identifies the top five differentially expressed genes (log2FC >2, adj. p<0.05) across the 11 clusters. G-H. UMAP plots of cells stratified by high (red) and low (blue) extracellular-related AUCell scores.

We further investigated the differences and significance of fibroblasts in PTC-blank-LNM and PTC-thyroiditis-LNM tissues at the single-cell level. Following stringent quality control, a total of 93,788 high-quality cells from 12 samples were retained for downstream analysis (**Supplementary Figures 1A, B**; **Supplementary Table 3**). After correcting for inter-sample batch effects using the Harmony method, UMAP visualization demonstrated that cells from different samples were well-integrated (**Supplementary Figure 1C**). All cells were partitioned into 48 clusters at a resolution of 1 (**Supplementary Figure 1D**). Unsupervised clustering and UMAP visualization using the Seurat workflow identified six major cell types (**Supplementary Figure 2**, **Figures 4A, B**), including thyroid epithelial cells(TG, CLU, FN1, MGST1, S100A13), B cells(CD79A, CD79B, MS4A1, IGKC, CD74), T and NK cells(CD3D, CD3E, IL7R, IL32, TRAC), myeloid cells(LYZ, FCER1G, LYZ, TYROBP), fibroblasts(RGS5, IGFBP7, TAGLN, COL1A2, ACTA2), and endothelial cells(TIMP3, RAMP2, CLDN5, TFPI, MGP). Compared to PTC-thyroiditis-LNM, the proportion of fibroblasts in PTC-blank-LNM tissues was significantly higher (**Figure 4C**). Subsequently, we isolated fibroblasts from PTC-blank-LNMs and performed dimensionality reduction and clustering (**Figure 4D**). These fibroblasts were classified into 11 subclusters, with the sixth subcluster highly expressing myofibroblast marker genes (**Figure 4E**). We calculated the signature genes for each fibroblast subcluster, and the sixth subcluster exhibited higher expression of PI16 and CHRDL1 (**Figure 4F**). Using AUCell, we scored the functions and pathways associated with extracellular matrix formation in each fibroblast. The sixth subcluster of fibroblasts was also significantly associated with ECM, regulating the extracellular matrix (**Figures 4G-H**). Therefore, our findings suggest that the sixth fibroblast subcluster may play a key role in regulating extracellular matrix components in PTC-blank-LNM tissues. To further characterize the PI16+ fibroblast subcluster, we first performed GO and KEGG enrichment analyses on its highly expressed genes (PI16+_fibroblast_genes). The results showed that PI16+ fibroblast was mainly associated with extracellular matrix remodeling (**Supplementary Figure 3A**). Protein-protein interaction (PPI) network analysis revealed that ECM genes, such as JUN, FN1, and COL1A1, were at the core of the network (**Supplementary Figure 3B**). Fibroblasts cell showed the strongest intercellular connections with thyroid epithelial cell in PTC-blank-LNM tissues (**Supplementary Figure 3C**). This connection was mainly manifested in the strong communication probability involving the ECM-receptor ligand pairs (**Supplementary Figure 3D**). Specifically, fibroblasts were closely linked to thyroid epithelial cells through the FN1 and COLLAGEN signaling pathways (**Supplementary Figures 3E-F**). In contrast, communication through these two pathways appeared less pronounced between other cell types and thyroid epithelial cells (**Supplementary Figure 3G**).

### Screening for PTC-LNM-related genes using machine learning

**Figure 5:**
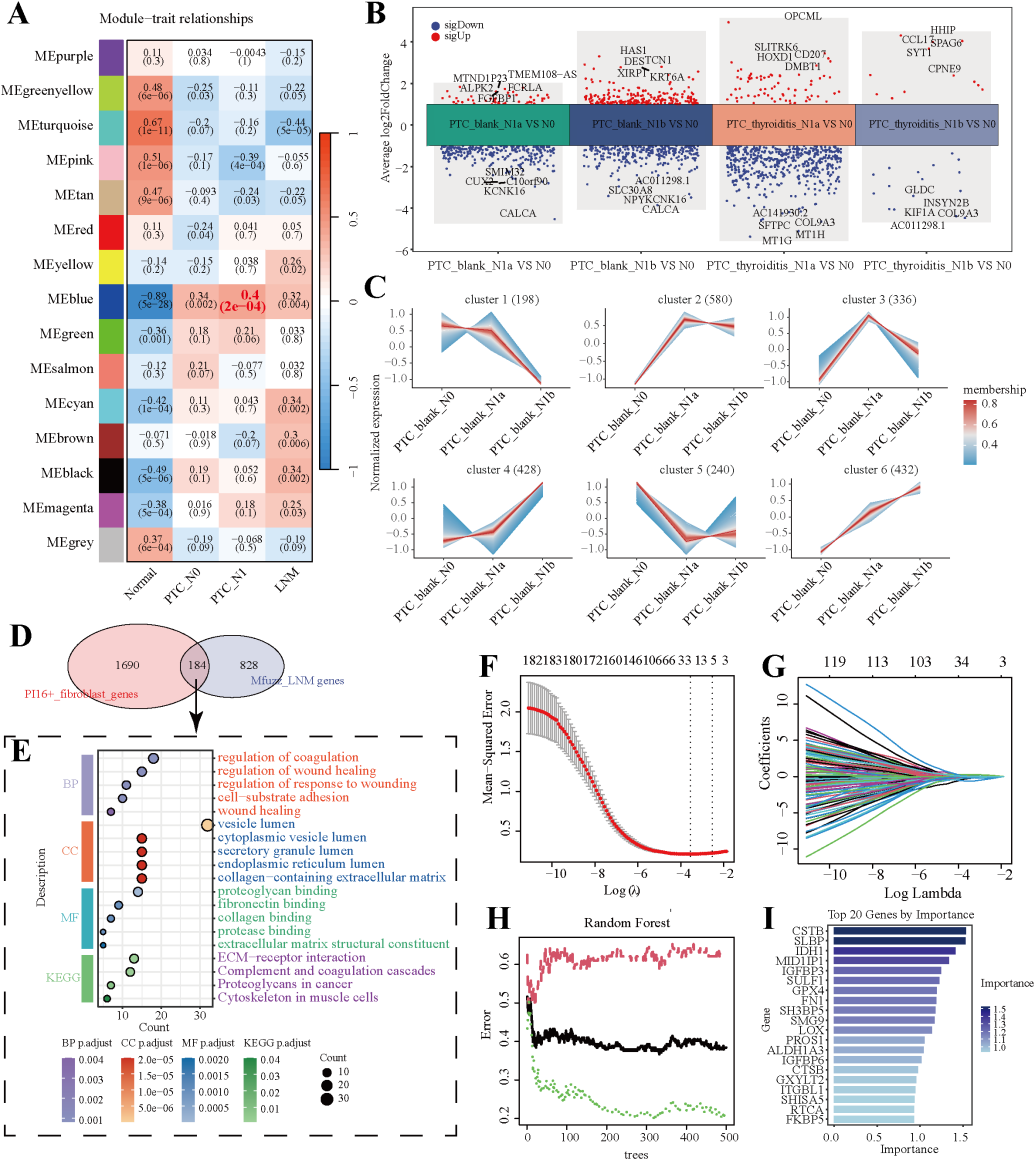
Screening of PTC-LNM-related genes. **(A)** DEGs between PTC-LNM and PTC non-LNM samples. The top five upregulated or downregulated DEGs are shown based on log fold change. **(B)** WGCNA results based on data from the four groups: Normal, PTC-N0, PTC-N1, and LNM. **(C)** Mfuzz clustering analysis of PTC-LNM progression. **(D)** Venn diagram of PTC-LNM-related genes identified through Mfuzz analysis and fibroblast-associated genes from cluster VI. **(E)** GO and KEGG pathway enrichment analysis of PTC-LNM-related genes. **(F, G)**. Screening results using the LASSO algorithm. **(H)** Relationship between the number of trees and error rates in the Random Forest model. **(I)** The top 20 genes are ranked by importance scores calculated by the Random Forest algorithm.

We performed WGCNA on the GSE60542 dataset to identify modules associated with LNM in PTC. This analysis revealed that the blue module, comprising 1,671 genes, showed the strongest correlation with PTC-LNM (**Figure 5A**; **Supplementary Figures 4A, B**). In parallel, using the TCGA database, we identified differentially expressed genes (DEGs) by comparing tissues from patients with PTC-blank-LNM and PTC-thyroiditis-LNM against those without LNM (**Figure 5B**). Applying a threshold of adjusted p-value (p_val_adj) < 0.05 and absolute average log_2_ fold change |avg_log2FC| > 1, we identified a total of 1,416 DEGs across the four comparison groups (**Supplementary Table 4**). Next, by integrating the 1,416 DEGs and the 1,671 genes from the blue module, we employed the Mfuzz algorithm to analyze the dynamic gene expression patterns during PTC-LNM progression. The results demonstrated that genes in clusters 2 and 6 exhibited a gradually increasing expression pattern from PTC-blank-N0 to PTC-blank-N1a and further to PTC-blank-N1b (**Figure 5C**). Therefore, we defined the genes contained in clusters 2 and 6 as Mfuzz-LNM genes. By intersecting the Mfuzz-LNM genes with the signature genes of the PI16^+^ fibroblast subpopulation, we identified 184 fibroblast-related LNM genes (**Figure 5D**). Functional enrichment analysis indicated that these genes were primarily associated with cell migration and extracellular matrix receptor signaling (**Figure 5E**). To refine this gene set for predictive modeling, we applied LASSO regression algorithm, which further filtered the 184 genes down to 21 core candidates. The variable selection process and cross-validation curve of the LASSO analysis are visually presented (**Figures 5F, G**). Independently, we also applied a random forest algorithm to rank the importance of the 184 genes, retaining the top 30% for downstream analysis (**Figures 5H, I**). Finally, taking the intersection of the gene sets identified by LASSO and random forest, we selected a consensus set of 17 genes to construct a predictive risk model for LNM in PTC patients (**Supplementary Figure 4C**).

### Construction and validation of a predictive model for PTC-LNM

**Figure 6:**
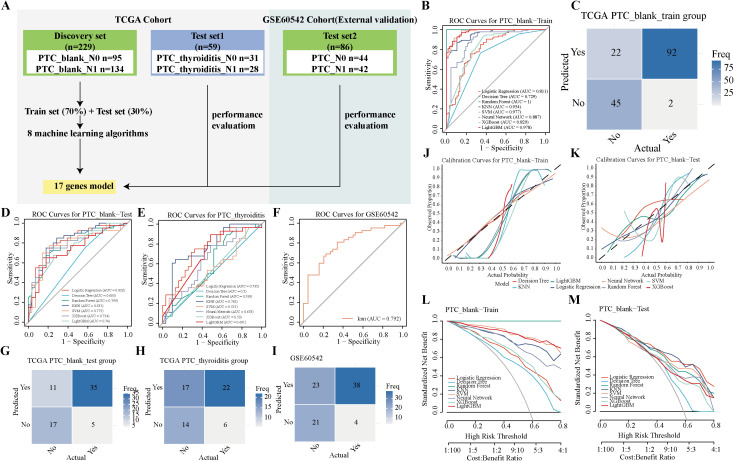
17-Gene model workflow and performance evaluation for PTC-LNM diagnosis. **(A)** Design of the modeling workflow. Eight machine learning algorithms were adopted for training predictive models using 17 selected genes. Model performance was validated in an internal test set and an external independent test set. **(B)** Receiver operating characteristic (ROC) curve for diagnosing PTC-LNM in the training set (TCGA-train dataset). **(C)** Confusion matrix illustrating prediction outcomes for the training set (TCGA-train dataset). **(D-F)**. ROC curves for diagnosing PTC-LNM in three test sets (TCGA-test, TCGA-thyroiditis and GSE60542 datasets). **(G-I)**. Confusion matrices of the prediction model in three test sets (TCGA-test, TCGA-thyroiditis and GSE60542 datasets). **(J, K)**. Calibration curves for diagnosing PTC-LNM in the TCGA-train dataset and TCGA-test dataset. **(L, M)**. Decision curve analysis (DCA) for diagnosing PTC-LNM in the TCGA-train dataset and TCGA-test dataset.

The samples used for model development and testing are illustrated in **Figure 6A**. Among the eight machine learning models evaluated, four achieved AUC values exceeding 0.9 in the training set (**Figure 6B**). The k-nearest neighbors (KNN) model emerged as the best performer, with AUCs of 0.831 in the test set, 0.762 in test set 1, and 0.792 in test set 2 (**Figures 6D-F**). After dataset correction, the model’s predictive efficacy on the test set and external validation set was confirmed (**Supplementary Figure 4D**). Confusion matrices further substantiated the high predictive accuracy of the KNN model (**Figures 6C, G–I**). Additionally, calibration curves (**Figures 6J, K**; **Supplementary Table 5**) and decision curve analysis (DCA) curves (**Figures 6L, M**) demonstrated the model’s robustness in both the training (AUC = 0.954) and test (AUC = 0.831) sets. SHAP swarm plots identified SULF1, S100A6, and LOX as the most influential predictors in the KNN model, with their high expression levels correlating with an elevated risk of metastasis (**Supplementary Figures 4E, F**).

### SHISA5 is a core molecule in PTC-LNM

**Figure 7:**
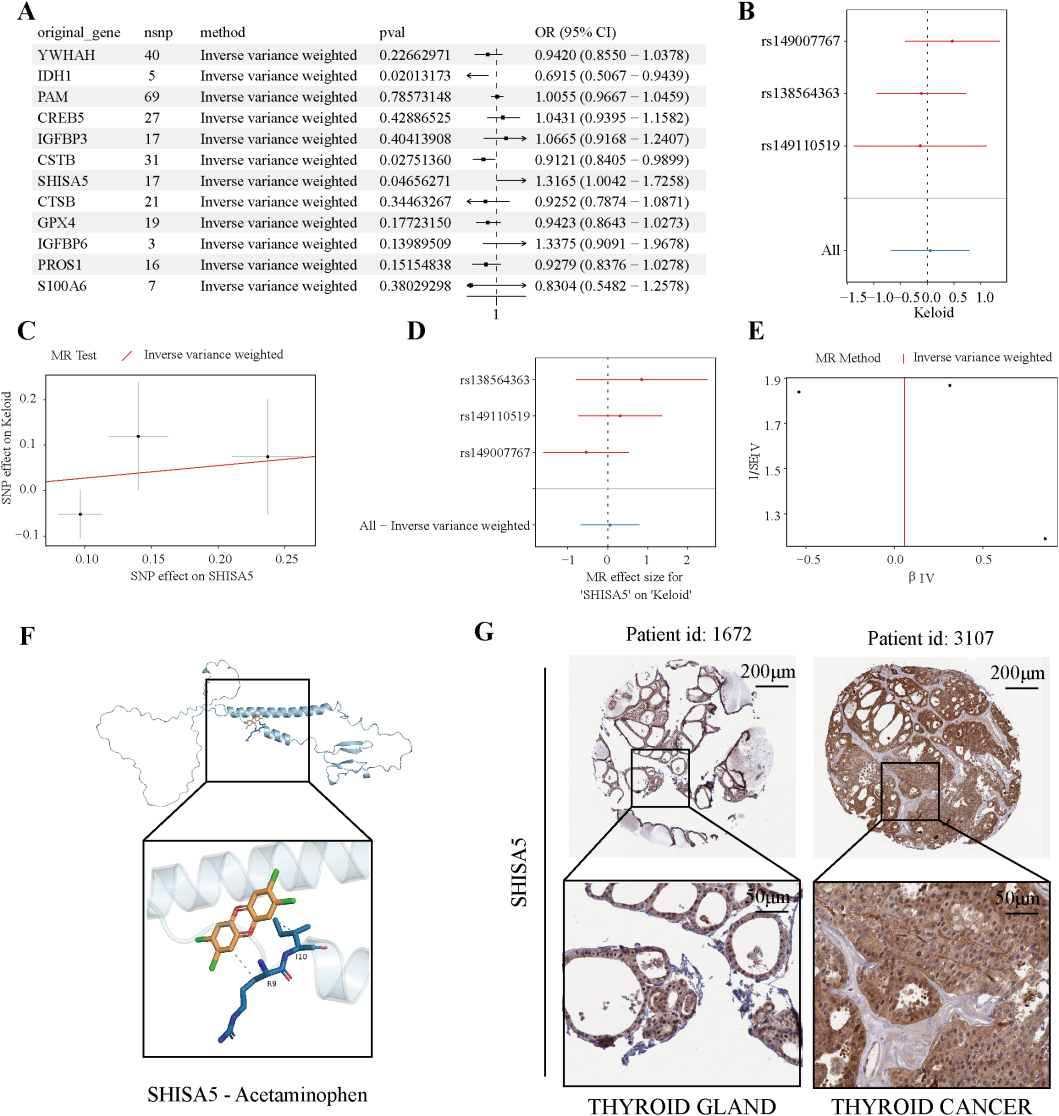
MR analysis and validation of core genes for PTC-LNM diagnosis. **(A)** Forest plot of the causal relationships between 11 Model genes and thyroid cancer. **(B)** Leave-one-out analysis of the causal relationships between SHISA5 and thyroid cancer. **(C)** Scatter plots of the causal relationships between SHISA5 and thyroid cancer. **(D)** Forest plot of the causal relationships between SHISA5 and thyroid cancer. **(E)** Funnel plots of the causal relationships between SHISA5 and thyroid cancer. **(F)** Molecular docking between Acetaminophen and SHISA5. **(G)** The expression levels of SHISA5 in thyroid and thyroid cancer tissue.

To investigate causal relationships between 17 candidate model genes and thyroid cancer, instrumental variables (IVs) were rigorously selected based on pre-defined inclusion criteria, resulting in 11 genes that met the thresholds. After excluding single nucleotide polymorphisms (SNPs) strongly associated with Alzheimer’s disease, 126 SNPs were retained, each exhibiting F-statistics greater than 10, confirming their robustness for Mendelian randomization (MR) analysis and minimizing weak instrument bias (**Supplementary Table 6**). Employing the inverse-variance weighted (IVW) method, we identified significant causal associations for specific genes: IDH1 demonstrated a protective effect [odds ratio (OR) = 0.6915, 95% confidence interval (CI): 0.5067–0.9439, P = 0.0201], along with CSTB (OR = 0.9121, 95% CI: 0.8405–0.9899, P = 0.0275), and SHISA5 (OR = 1.3165, 95% CI: 1.0042–1.7258, P = 0.0466), indicating their roles as protective factors against thyroid cancer risk (**Figure 7A**). To comprehensively validate the causal link involving SHISA5 and assess result stability, extensive sensitivity analyses were conducted (**Supplementary Tables 7**–**9**). Leave-one-out analysis confirmed that the positive association was not unduly influenced by any single IV, eliminating concerns of bias from outlier variants (**Figure 7B**). Scatter plots clearly depicted a positive correlation between weighted genetic risk scores and thyroid cancer incidence (**Figure 7C**), while forest plots reinforced consistent directional effects across instruments (**Figure 7D**). Funnel plots revealed no significant asymmetry, suggesting minimal heterogeneity and low potential for directional pleiotropy (**Figure 7E**), further corroborating SHISA5’s role as a causal risk factor. Exploration of SHISA5’s therapeutic relevance through molecular docking identified high binding affinity with acetaminophen (paracetamol), indicating its potential as a druggable target (**Figure 7F**). Tissue-level expression analysis showed significantly elevated SHISA5 levels in thyroid cancer versus normal tissues (**Figure 7G**), providing biological validation. Collectively, this multi-faceted approach—integrating genetic causality inference, sensitivity robustness, target discovery, and expression corroboration—confirms SHISA5 as a causal gene in thyroid cancer pathogenesis, with acetaminophen implicating a plausible intervention pathway.

## Discussion

Consistent with the previous studies (28), our findings showed that patients with PTC without thyroiditis had worse TNM staging of the tumor. The BRAF gene is the most common mutation site in patients with PTC, and BRAF mutations usually indicate that patients with PTC are more likely to experience LNM and may have a worse prognosis (23, 29). Compared with PTC thyroiditis, we observed a higher proportion of BRAF gene mutations in PTC-blank. A recent study highlighted the importance of MUC16 mutations in PTC, with MUC16 mutations being closely associated with higher recurrence risks, more frequent LNM, and higher T stages in patients with PTC (21). Similarly, we found that patients with PTC-blank had a higher probability of MUC16 mutations than patients with PTC-thyroiditis. The higher proportion of BRAF and MUC16 gene mutations in PTC-blank may be related to their worse prognosis. Consistent with previous studies, T and B cells showed the most significant differences in infiltration between PTC-blank and PTC-thyroiditis tissues (30, 31).

In addition to BRAF gene mutations (32), current perspectives on the mechanisms of PTC-LNM mainly include abnormal expression and modification at the molecular level (33, 34), abnormal infiltration of immune cells into tumor tissues (35, 36), and alterations in extracellular matrix components (37, 38). Collagen is a major components of the ECM, and its dynamic remodeling is thought to promote metastasis in various cancers by affecting the physicochemical properties of the tumor extracellular matrix (39–41). Enrichment analysis results showed significant activation of functions related to the extracellular matrix remodeling process, such as peptide cross-linking and collagen catabolism, during the PTC-blank-LNM process. We further analyzed the changes in the expression of extracellular matrix-related markers (ITGB1, LOX, and POSTN) during PTC-LNM (42–44). The results showed that ITGB1, LOX, and POSTN were significantly upregulated in PTC-blank-LNM, a phenomenon not observed in PTC-thyroiditis-LNM. Sirius Red staining also showed a more significant increase in collagen fiber content in PTC-blank-LNM tissues than in PTC-thyroiditis tissues. Cancer-associated fibroblasts have been shown to regulate collagen cross-linking and degradation by secreting LOX and matrix metalloproteinases, thereby affecting the invasiveness and migratory ability of tumor cells (45, 46). Previous studies have identified the recruitment of fibroblasts to the TME as a key feature as a feature associated with thyroid cancer progression (47, 48). Using various immune scoring algorithms, we also found an increase in the proportion of fibroblasts during the PTC-blank-LNM process; however, no increase in CAFs was observed in PTC-thyroiditis-LNM tissues, a phenomenon not previously reported. Future studies should explore this finding using large-scale sequencing and clinical samples. Subsequently, we identified a population of fibroblasts with high PI16 expression in PTC-blank-LNM tissues, which exhibited a higher score for extracellular matrix remodeling. In esophageal squamous cell carcinoma (ESCC), fibroblasts with high PI16 expression promote LNM and exhibit poor overall survival and cisplatin resistance (49). However, some studies have indicated that PI16+ fibroblasts may have potential antitumor functions in adjacent non-cancerous regions (50), which may be related to cellular heterogeneity in different tissues. Therefore, compared with PTC-thyroiditis, higher BRAF and MUC16 gene mutations and extracellular matrix remodeling are associated with PTC-blank to be more prone to LNM.

By integrating bulk RNA-Seq and scRNA-seq to screen for LNM-related molecules, we developed a 17-gene signature model to diagnose LNM in thyroid cancer. This model exhibited high specificity and sensitivity for multiple datasets. Through SHAP values and clinical significance, this study identified that LOX contributes more to PTC-LNM and further validated this through IHC of clinical samples. LOX family proteins consist of five paralogs: LOX and LOX-like 1-4 (LOXL 1-4), characterized by catalytic extracellular matrix cross-linking remodeling (51). Dysregulation and abnormal expression of LOX family proteins are associated with the occurrence and progression of various human cancers, including lung, hepatocellular, gastric, renal cell, and colorectal cancers (52). Previous studies have already found upregulation of COL1A1 and LOX in PTC tissues (47, 48). Compared with PTC without LNM, LOX expression was further upregulated in PTC-LNM tissues, further suggesting a potential role of LOX in the PTC-LNM process.

The strong interaction between SHISA5 and acetaminophen, as shown by molecular docking, and the causal role of SHISA5 confirmed by MR, hint at acetaminophen’s potential therapeutic effects. SHISA5 belongs to the Shisa protein family, characterized structurally by single transmembrane domains with cysteine-rich motifs at the N-terminus and proline-enriched segments at the C-terminus. This protein exhibits dual localization in both the endoplasmic reticulum and nuclear membrane. Functionally, SHISA5 participates in multiple cellular processes, particularly in modulating autophagy pathways. It contributes to p53-mediated apoptotic responses through caspase-dependent mechanisms and demonstrates responsiveness to interferon stimulation. Additionally, SHISA5 exerts regulatory effects on Wnt signaling cascades (53, 54). SHISA5, identified as a potential biomarker here, may be involved in the disease - related pathways. Acetaminophen, a commonly used drug, could potentially modulate the function of SHISA5 and thereby influence the disease process. This finding not only provides a new avenue for drug repurposing but also suggests that SHISA5 could be a therapeutic target.

This study has certain limitations that warrant consideration. First, while our integrative multi-omics approach reveals strong associations between fibroblast infiltration, ECM remodeling gene signatures, and LNM in PTC-blank, we acknowledge that the present study primarily demonstrates correlation rather than mechanistic causation. The observed enrichment of ECM-related pathways, increased fibroblast proportions, and collagen deposition in metastatic samples suggest but do not prove a direct causal role. Future functional studies using *in vitro* and *in vivo* models are needed to experimentally validate whether PI16+ fibroblasts actively drive ECM remodeling that promotes LNM in PTC-blank. Second, the retrospective nature of public datasets constrained comprehensive clinicopathological variable control; *in vitro* validation of SHISA5-acetaminophen interactions remains pending; and fibroblast subcluster functions necessitate lineage-tracing validation. To address these gaps, future studies should explore thyroiditis-induced immune checkpoint alterations, develop SHISA5-targeted nanotherapeutics leveraging acetaminophen’s binding affinity, and prospectively validate the 17-gene model in multicenter cohorts to establish clinical utility.

By integrating bulk RNA-Seq and scRNA-seq in this study, we identified associations between the impact of cancer-associated fibroblasts, and collagen on PTC-LNM and further observed that this mechanism primarily occurs in patients with PTC-blank. In addition, we constructed a model to predict PTC-LNM. This study systematically analyzed the molecular and cellular heterogeneity of PTC-blank-LNM and PTC-thyroiditis-LNM, providing valuable guidance for the clinical management of patients with PTC, with or without thyroiditis.

## Data Availability

The data presented in the study are deposited in the Gene Expression Omnibus (GEO) and UCSC Xena repositories. The GEO accession numbers for the datasets are GSE138198, GSE60542 and GSE184362. The UCSC Xena data can be accessed via the UCSC Xena browser (https://xenabrowser.net/datapages/).
