# Supplementary figures and images for: Integrating multi-omics and machine learning to unravel mechanisms of lymph node metastasis in papillary carcinoma with and without thyroiditis
Source: Front Immunol. 2026 Feb 18;17:1708330. doi: 10.3389/fimmu.2026.1708330 (PMC12957244; doi:10.3389/fimmu.2026.1708330)

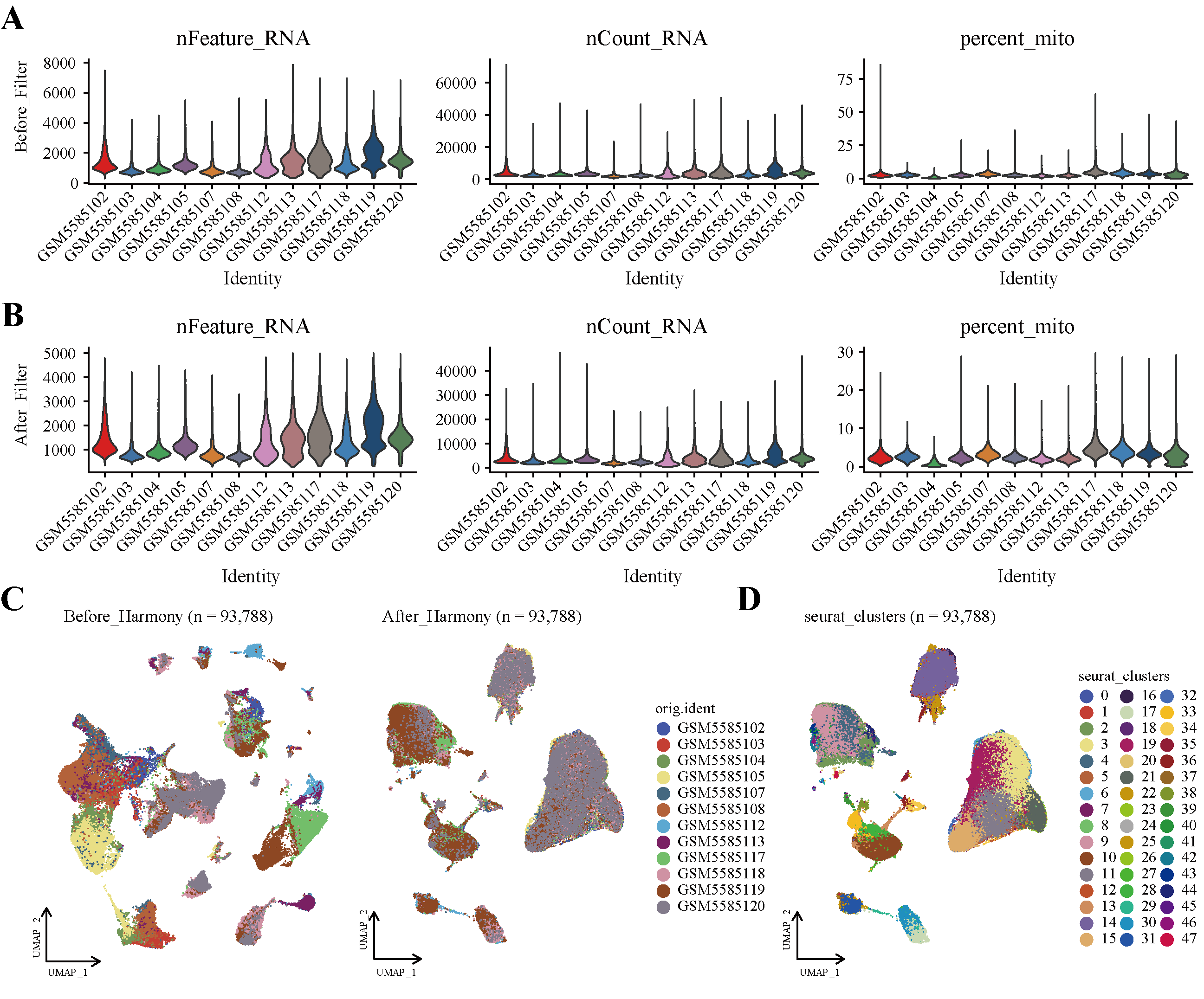

Supplement: Supplementary file 1 [file Image1.tif]

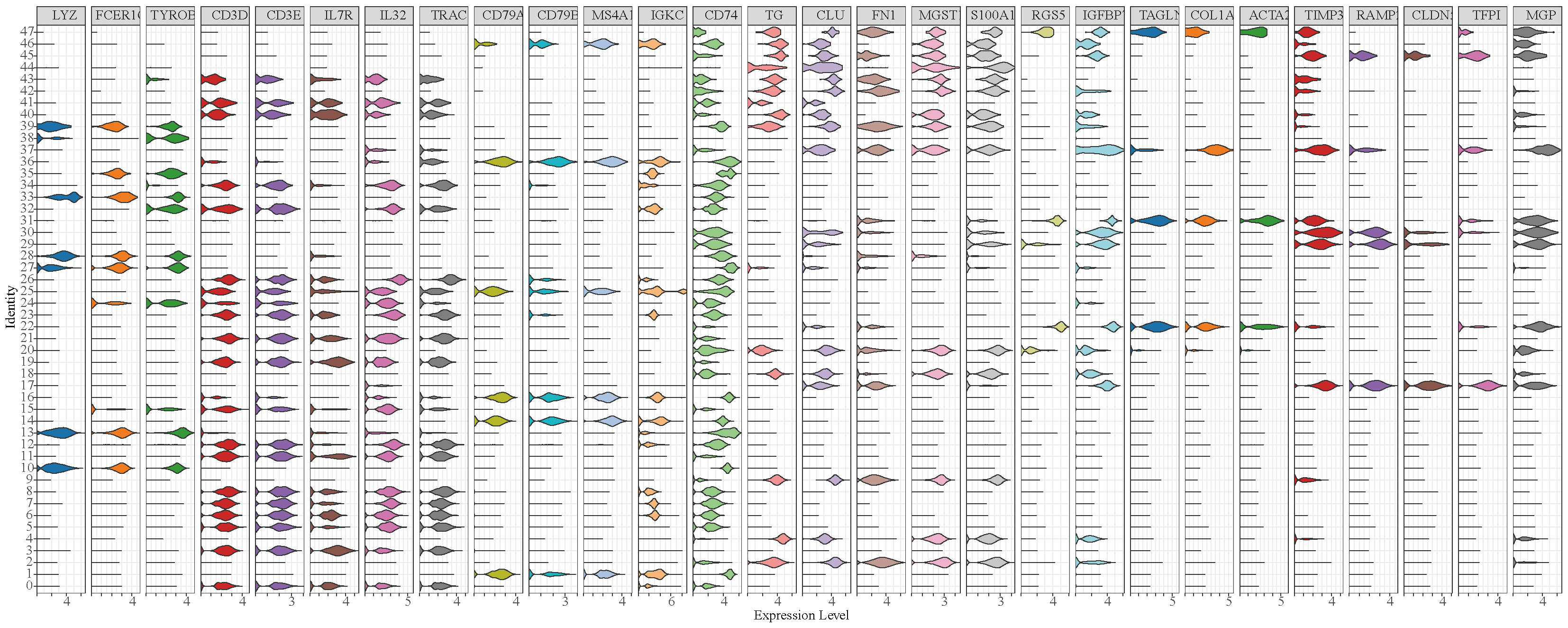

Supplement: Supplementary file 2 [file Image2.tif]

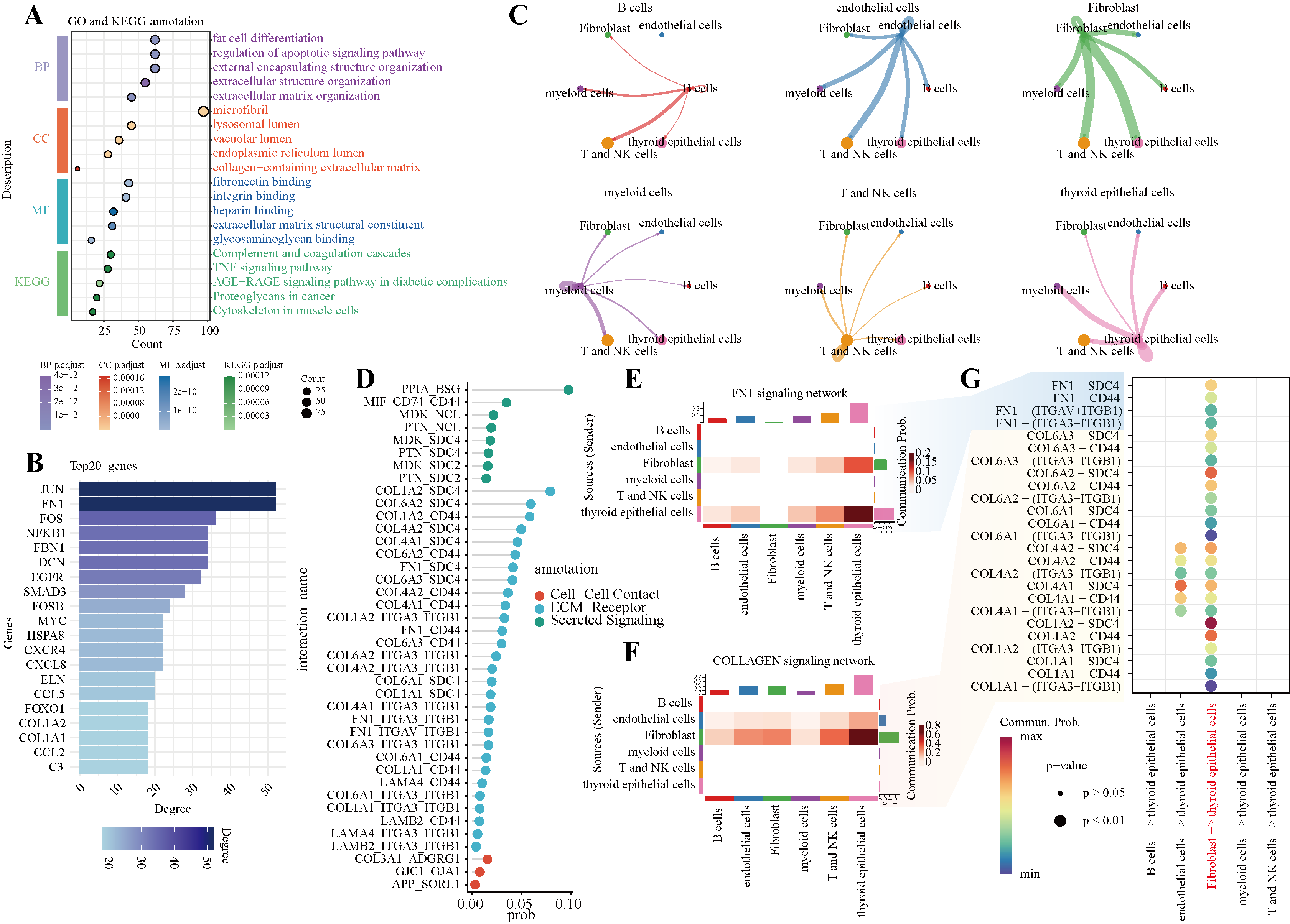

Supplement: Supplementary file 3 [file Image3.tif]

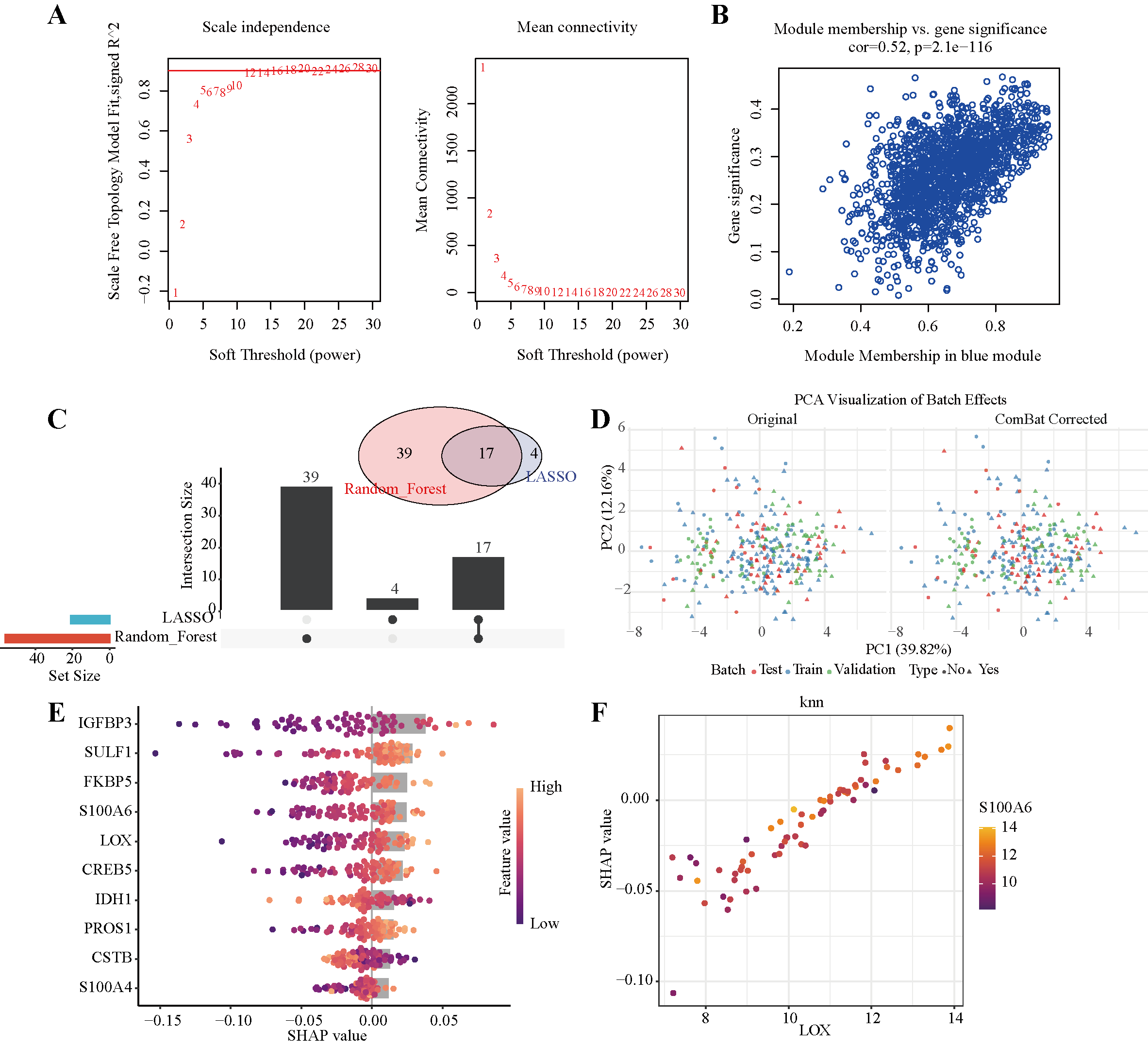

Supplement: Supplementary file 4 [file Image4.tif]
